# Waste metrics in the framework of circular economy

**DOI:** 10.1177/0734242X231190794

**Published:** 2023-08-21

**Authors:** Irene Voukkali, Iliana Papamichael, Pantelitsa Loizia, Demetris F Lekkas, Teresa Rodríguez-Espinosa, Jose Navarro-Pedreño, Antonis A Zorpas

**Affiliations:** 1 Laboratory of Chemical Engineering and Engineering Sustainability, Faculty of Pure and Applied Sciences, Open University of Cyprus, Latsia, Nicosia, Cyprus; 2 Waste Management Laboratory, Department of the Environment, University of the Aegean, Mytilene, Greece; 3 Department of Agrochemistry and Environment, University Miguel Hernández of Elche, Elche (Alicante), Spain

**Keywords:** Circular economy, sustainable development goals, waste management, waste metrics

## Abstract

There are several sustainability issues that the linear economy of today’s society cannot adequately tackle (i.e. resource depletion, waste treatment, etc.). As a result, the scientific community and policymakers give high priority to the implementation of the circular economy concept. The sustainable development goals of the United Nations are in line with the European Union’s (EU) commitment to a smooth transition to a circular economy. Circular business models require a shift in technical elements involving R strategies to replace traditional business models (i.e. reuse, reduce, recycle, etc.). Monitoring circular economy to provide quantifiable, measurable data is necessary for a successful transition. Monitoring tools (i.e. Key Performance Indicators, quality protocols) enable decision-makers to measure circular economy performance and identify circularity’s advantages and disadvantages. To stimulate the adoption of a circularity model addressing critical issues of excessive waste production and resource use, this mini review aims to address the literature gap of waste metrics in the framework of circular economy and offer insights on circular economy indicators to aid for a seamless transition to a more sustainable society. For this purpose, Preferred Reporting Items for Systematic Reviews and Meta-Analysis method was chosen to assess literature. The authors collected and analysed data from 101 records, 70 articles and 31 reports related to the topic under consideration. Through the literature review, it is obvious that moving away from linear production model frequently leads to the development of new internal capabilities along the value chain and, eventually, high efficiency that reduces costs, increasing productivity, encourages brand names, minimizes threats, creates new products and fulfils regulations and green consumer expectations.

## Introduction

**Figure 1.**
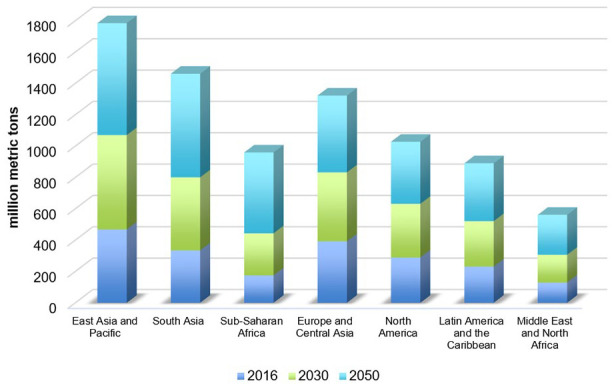
Projection of waste generation worldwide in 2016, 2030 and 2050, by region (in million metric tonnes) (Statista, 2023).

Environmental pollution and resources scarcity are among the major challenges of sustainability in today’s world, as a result of urbanization and industrialization over the last decade (Hoang et al., 2022). Increased human needs result in overconsumption of natural resources, leading to increased environmental pollution such as water pollution, air pollution and land pollution, decreasing quality of life and restricting economic growth (Wang and Li, 2021; Zhang et al., 2022).The rapidly growing human population, which is expected to reach 10 billion people by 2050, is also considered a threat that would exacerbate the current situation even further (Worldometer, 2022). The existing linear economy model tends to overuse natural resources and produce a significant amount of waste (UNDP, 2019). Unrelenting population explosion in combination with unsustainable consumption behaviour produces even more waste, which increases direct and indirect environmental challenges (European Environment Agency, 2014; Levaggi et al., 2020). If humanity continues on its current path consumption pattern, the resources of three Earths will be required to meet human needs by 2050 (Voukkali and Zorpas, 2022). The current resource usage pattern produces five tonnes of waste per European citizen yearly (European Commission, 2021a). Global waste production is expected to continue rise over the coming decades. Increasing amount of waste combined alongside with poor waste management systems results in climate change, air water and soil pollutions, biodiversity losses and others (Marques and Teixeira, 2022). In 2016, the total waste generation in East Asia and the Pacific amounted to 468 million metric tonnes, while the quantities produced in other continents were also increased. Forecasts show that by 2050, waste production in this region will increase to 714 million metric tonnes (Figure 1). Since waste generation is generally expected to increase in tandem with economic expansion and population growth, nations with high proportions of growing low-income and lower-middle-income countries are expected to be the most affected. With economic growth and urbanization, waste levels in Sub-Saharan Africa and South Asia are predicted to triple and double, respectively, during the next three decades. On the other hand, waste levels are predicted to rise more gradually in regions with higher-income countries, such as North America, Europe and Central Asia. More specific daily per capita waste production in high-income countries is forecast to rise by 19% by 2050, whereas it is projected to rise by 40% or more in low- and middle-income countries (The World Bank, 2018).

According to the World Bank (2022), roughly 2.2 billion metric tonnes of waste were generated worldwide in 2020, and this number is expected to rise to 3.88 million by 2050. Approximately 70% of municipal solid waste collected by cities ends up in landfills, 19% is recycled, and 11% is used for energy recovery. The waste recycling rate in the EU-27 is increasing, as a result of a rising number of globally initiatives and efforts aimed at changing daily habits including the promotion of prevention, reuse, repair, recycling, etc. Considering the statistics above, it is obvious that waste will continue to increase on the entire planet, and there is no doubt about it (Zorpas, 2020).

The circular economy concept strives to transform traditional practices of production and economic growth, which are considered as linear systems, into circular dynamics that connect resource use and waste generation to reduce pollution and waste production (Buchmann-Duck and Beazley, 2020; Wang et al., 2020). In response to global environmental degradation and the imperative need for change, the idea gained acceptance all over the world, resulting in the implementation of national strategies and policies based on the principle of a circular economy (Apolloni et al, 2021; Kara et al., 2022; Morseletto, 2020). According to Provin et al. (2021), circular economy is a regenerative system in which resource flows are significantly reduced aiming at long-term projects with a focus on maintenance, repair, reuse and recycling strategies. Furthermore, the circular economy can encourage the long-term support for development by decoupling economic growth from the negative effects related with depletion and degradation of environment (Durán-Romero et al., 2020). As a result, circular economy provides the opportunity to link eco-efficiency and profit growth (Kurdve and Bellgran, 2021). The need to achieve sustainable development goals (SDGs), strengthen resource efficiency and end planned obsolescence has fuelled the appeal of transitioning to a circular economy (Kara et al., 2022; Mies and Gold, 2021). Sustainable consumption and production (SDG 12), clean water and sanitation (SDG 6), clean and available energy (SDG 7), climate change (SDG 13), SDG 12 (responsible production and consumption), Life Below water (SDG 14) and Earth’s life (SDG 15) are all closely linked to circular economy practices (Centobelli et al., 2020; Kristoffersen et al., 2020; UNDP, n.d.). While circularity has a clear link to the above SDGs, particularly those in the environmental or economic domains, its link to others also hints at immense potential: when used holistically, a global circular economy can drive SDG attainment. These goals are: No poverty (SDG 1), Zero hunger (SDG 2), Good health and wellbeing (SDG 3), Gender equality (SDG 5), Reduced inequalities (SDG 10) and Peace, justice and strong institutions (SDG 16) (Schroeder et al., 2019). Beyond material usage, the circular economy has the potential to improve practices that generate greenhouse gases and pollute environment, including land, water and air. It can also help create a more just, equitable world by providing more equal access to resources, equity among minorities and a range of safe, decent jobs if applied holistically (Puntillo, 2023).

This mini review intends to provide insights on waste key performance indicators (KPIs) in the framework of circular economy for the adoption of a circularity model that encourages the approach to crucial concern of over-exploitation of natural resources and excessive waste production. The exploration of waste monitoring specific to circular economy in literature is deficient, whereas a collective evaluation of all existing tools for measuring circular economy will be deemed crucial for a smooth transition towards a more sustainable society.

### Theoretical background

The European countries that have achieved significant results in implementing the principles of the circularity approach are mainly characterized by their continued attempts to transition to a circular economy, as well as their targets and goals. Given the several principles of circular economy, and how diversely one single nation could indeed score on each of these concepts, it still is challenging to precisely classify how countries achieve in terms of circular economy. Germany leads in certain aspects of circular economy, such as waste management and recycling (Eurostat, 2021). France is now also trying to add numerous strategies and initiatives to promote a better circular economy model in the country, including the circular economy roadmap for 2018, which includes 50 measures (Ministry for an Ecological and Solidary Transition, 2018). The French roadmap divided into four key priority areas: (i) better production, (ii) better consumption, (iii) better waste management and (iv) increased participation of all stakeholders (FREC, 2018). Belgium also serves as a considerable player in the field. It ranked second in the rate of circular material use, trailing only France and the Netherlands. Austria, Italy, Denmark and Slovenia are also noteworthy countries (Eurostat, 2021). Beyond the EU, countries, such as China, Japan, Brazil, Canada and the United States, are attempting to achieve the transition. Most countries in the lead in the field of circular economy are EU countries, implying that Europe as a whole is currently in the lead group (Ellen MacArthur Foundation, 2021).

The European Commission’s initiatives including European Green Deal (EGD), Fitfor55 strategy, Zero Pollution Act, European Industrial Strategy, etc. are also noteworthy, with concerning a state of play for a global and circular economy and the new Circular Economy Action Plan (CEAP) being one of the main blocs of the green deal (European Commission, 2020, 2021a, 2021b). Even though the EU in its entirety is a powerful player actor, a few European countries are still having trouble accelerating the transition. These countries are mostly from Eastern Europe including Bulgaria, Romania, Hungary, Slovakia, but also Greece, Portugal, Croatia and even Germany in specific fields.

**Figure 2.**
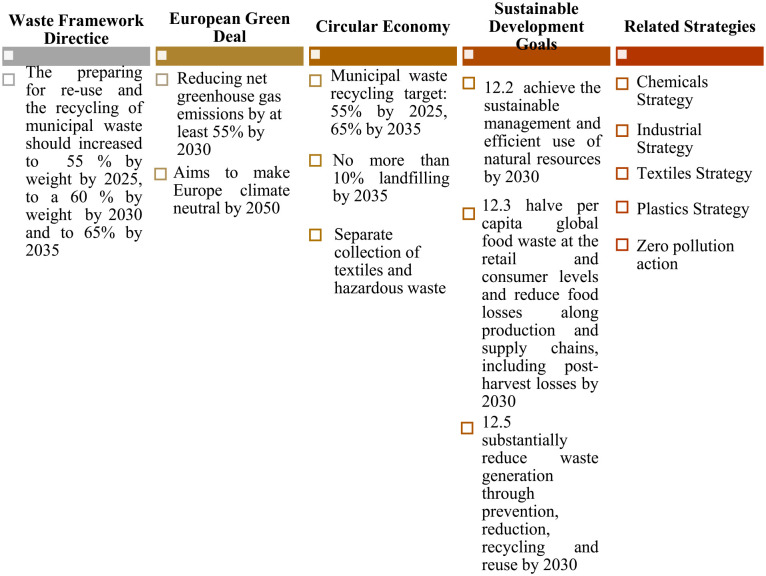
Legislative and non-legislative initiatives and targets for waste production and management.

In the context of current environmental, social and economic issues, many efforts have been taken in global and European level in order to identify and implement efficiency actions through the establishment of legislated and non-legislative initiatives, setting specific goals and targets (Figure 2). Up until 2008, the EU published approximately 1200 documents including the Waste Framework Directive (WFD), which established the fundamental principles of waste management (European Commission, 2008). WFD compelled member countries to develop numerous strategies, with a focus on prevention, reuse, reduction, recycling and energy recovery. Similarly, the WFD establishes some essential waste management concepts, in which waste management takes into consideration the human health and environmental protection. Furthermore, WFD presented the Polluter Pays Principle and the Producer Responsibility principle (Zorpas, 2020).

Later on in 2015, the United Nations published the 2030 Agenda for Sustainable Development, which includes 17 SDGs and 169 targets that can be measured using 230 specific qualitative and as well as quantitative indicators (Fuldauer et al., 2022; Priyadarshini and Abhilash, 2020). Regarding waste production and management, the UN SDG 12 set a specific deadline of 2030 to decrease losses and waste production through the implementation of prevention, reduction, recycling and reuse, along with a goal to motivate enterprises to adopt sustainability initiatives (United Nations, 2015).

Further on, the EGD was approved by the European Commission, in 2019. It introduced a roadmap attempting to make the EU’s economic system sustainable by transforming climate and environmental issues into opportunities in all policy priorities. The EGD aims to increase resource efficiency by transitioning to circular economy, as well as to inhibit climate change, reverse biodiversity loss and reduce pollution (D’ Adamo et al., 2023). The objectives encompass, along with other (i) a 90% lowering in transportation emissions, (ii) a GHG reduction target for 2030 of at least 50% and up to 55% in comparison to 1990 levels, (iii) zero-carbon steel by 2030 and (iv) the promotion of circular economy (European Commission, 2019). In July 2021, the EC adopted ‘Fit for 55’, a package of policy initiatives preparing for the EGD’s implementation. The term ‘Fit for 55’ refers to the EU’s goal of minimizing net GHGs emissions by at least 55% by 2030. The proposal package seeks to provide an integrated and balanced framework for achieving the EU’s climate objectives, which (i) assures an equal and socially fair transition, (ii) retains and strengthens EU industry’s creativity and competitiveness while maintaining a level playing field with third-country economic operators and (iii) supports the EU’s position as a global leader in the fight against climate change (European Commission, 2021b). In addition to the aforementioned actions, the EU has developed a plethora of waste-related strategies, related with specific waste streams (plastics, textiles, etc.) (European Commission, 2019).

### Circular economy action plan

**Figure 3.**
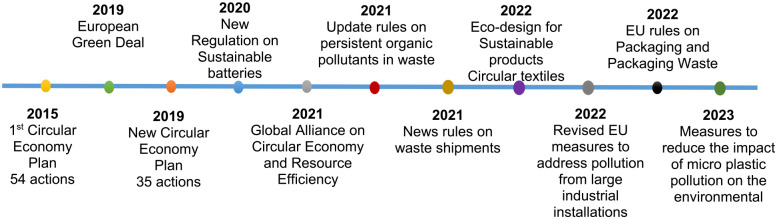
Timeline: Previous and upcoming actions and initiatives from EU for circular economy.

The first circular economy action plan was finalized in 2019, and its 54 actions have been completed (European Commission, 2015). Later on, a new Circular economy plan took place with 35 new actions (Figure 3). In March 2022, in line with the EU’s Green Deal climate neutrality goal of 2050 (European Commission, 2019), the European Commission proposed the first package of measures to speed up transition towards a circular economy, in line with CEAP. The proposals promoted sustainable products, empowering consumers for green transition, reviewing the construction product regulation and developing a sustainable textiles strategy. Furthermore, the European Commission published new EU packaging rules. It contained recommendations for improved design concept, including clear labelling, to encourage recycling and reuse. It also endorses the use of bio-based, degradable and compostable plastics. In addition to the modifications mentioned above, new amendments took place related to persistent organic pollutants in order to reduce the amount of dangerous chemicals in waste and manufacturing processes. The new guidelines imposed new restrictions, prohibited the use of certain chemicals and kept pollutants out of recycling. Waste prevention, eco-design and re-use could provide financial benefits for EU organizations while also lowering yearly Greenhouse gasses (GHGs) (D’Adamo et al., 2022; Papamichael et al., 2022a).

Nowadays, 45% of CO_2_ emissions are linked to the manufacturing of materials used every day. Transitioning towards a circular economy model may provide advantages such as reducing environmental pressures, improving raw material supply security, enhancing business competitiveness, enhance economic growth (an extra added 0.5% of GDP) and creating more jobs (700,000 jobs in the EU 2030). Consumers will additionally have more long–lasting and innovative products that will improve their life quality (European Commission, n.d.).

### Measuring circular economy progress

The circular economy gained more attention from policymakers, academics and researchers, as a result of the increase frameworks, strategic plans and legislation of the EU. Despite the fact that circularity has emerged as a major topic, there remains a scarcity of knowledge in the literature on tracking progress towards it (De Pascale et al., 2021). A comprehensive study on the progress towards the circular economy model that preserves a balance of economic, social and environmental aspects is missing from the circular economy literature (Tan et al., 2022). The absence of agreement among scientists and policymakers on guidelines for assessing progress towards circularization makes this process more difficult and unfamiliar (Giannakitsidou et al., 2020). Recognizing the economic forces of circular economy implementation is required for the development of more targeted strategies and policies, which will seek to assess the level at which the circular economy, has been fulfilled by establishing specific objectives, indicators and actions (D’Adamo et al., 2022; Marques and Teixeira, 2022). Even though numerous methods have been used to evaluate the transition to a circular economy, especially related with waste production and management, still, there is no standard tool that addresses all of the under-study concerns. The most commonly used tools for monitoring Circular Economy and environmental performance in general are: (i) KPIs, (ii) Life Cycle Assessment (LCA), (iii) Multi-criteria analysis, (iv) Material Flow Analysis (MFA), (v) Environmental Management Systems (i.e. ISO 14001, EMAS) and (vi) Digitalization (D’Adamo et al., 2022; Millette et al., 2019; Pappas et al., 2022; Voukkali and Zorpas, 2022; Wang et al., 2022).

Circularity KPIs are classified based on parameters such as the level of circular economy implementation, the circular economy loops, the efficiency and the perception of circularity (Rincón-Moreno et al., 2021). Due to the complexity of circular economy monitoring, the majority of the circularity indicators have been criticized for failing to capture the circular economy’s wider context, as they need to be interconnected according to circularity of the given subject matter (city, industry, etc.). This underlines a restriction to thoroughly assessing information about circular economy strategies (Papamichael et al., 2023b). Consequently, the methods and criteria developed for assessing the level of circularity for products, organizations or provinces do not adhere to a widely known set of guidelines (Rincón-Moreno et al., 2021; Saidani et al., 2019).

Simultaneously, policymakers and stakeholders have implemented quality protocols that provide standardization in the measurement and evaluation of KPIs. Quality protocols are being implemented to guarantee the safety, quality and service efficiency taking into consideration all the three pillars of sustainability and circular economy goals (Loizia et al., 2021). ISO 37101 and ISO 37120 are common protocols used in urban settings for defining and measuring the implementation of SDGs in and Circular Economy. ISO 37101 involves a wide range of stakeholders, which fosters synergy and a clear understanding of the objectives (White, 2021). Additionally to the above, other protocols are also used such as Environmental Management System (ISO 14001, EMAS, etc.) taken into consideration, eco-design, environmental costs and benefits, circulation, material flow cost and others (Loizia et al., 2021).

Notwithstanding, due to today’s technological advancements and innovation, these qualitative and quantitative tools all are covered by the umbrella of digitalization (Beliatis et al., 2019). The concept entails the incorporation of technology into daily life, assisting in providing real-time results, monitoring and problem-solving by eliminating external and internal errors caused by human intervention on a large scale (Mondejar et al., 2021). The endorsement of smart system development could assist authorities and policymakers address shared challenges of the circular economy and come up with novel strategies. Concepts, such as the Internet of Things (IoT), geographic information systems, benchmarking, artificial intelligence and many others, can catalyse innovation opportunities in the circular economy and sustainability. Furthermore, digitalization combined with other concepts may be utilized to pique the interest of policymakers, researchers and authorities in order to engage in active actions according to EU legislation (Massoud et al., 2021; Papamichael et al., 2022b; Roy et al., 2021).

## Methodology

**Figure 4.**
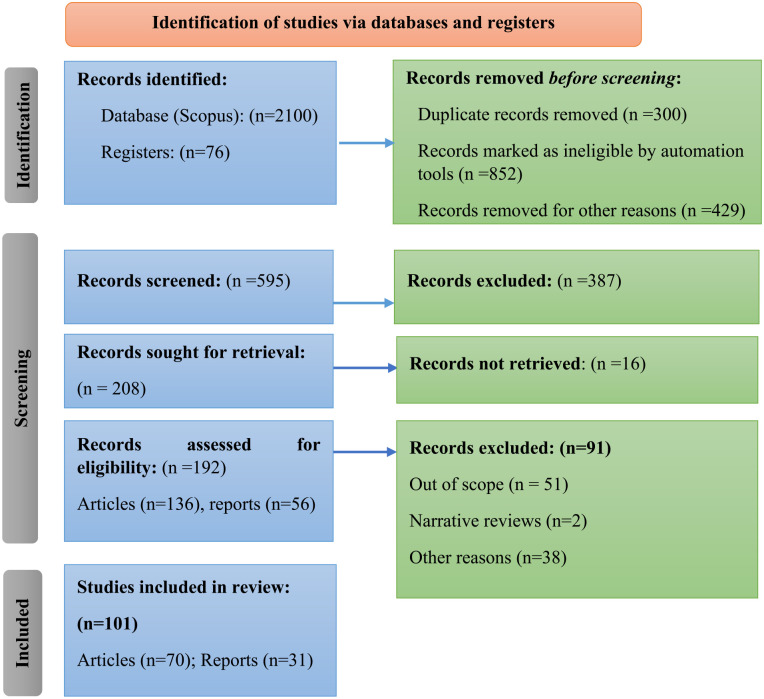
PRISMA 2020 flowchart for systematic reviews that involves searches of databases and registers exclusively. PRISMA: Preferred Reporting Items for Systematic Reviews and Meta-Analysis.

The Preferred Reporting Items for Systematic Reviews and Meta-Analysis (PRISMA) method was chosen to assess the state of the art (Figure 4). The proposed literature was reviewed using the PRISMA process, which involves 27 routes and encloses the well-defined stages of a systematic review, including eligibility criteria and relevant information sources, strategy exploration, selection procedure, results and data analysis (Page et al., 2021; Rethlefsen et al., 2021; Tricco et al., 2018; Voukkali and Zorpas, 2022). The PRISMA 2020 checklist consists of seven sections and topics (Title, Abstract, Introduction, Methods, Results, Discussion and Other Information) as well as 27 sub-criteria that should be met.

Both inclusion and exclusion criteria were used as eligibility criteria. The inclusion criteria include the following: (i) research related with key performance indicators, circularity waste production and management; (ii) published articles and reports from 1990 to the present; (iii) review papers; (iv) methodical demonstration and synthesis of research results and (v) records identified using the authors’ keywords. The exclusion criteria include the following: (i) narrative reviews and (ii) available papers in languages other than English. For the literature review, the database of Scopus was preferred. As Scopus database option search were ‘title, abstract, keywords’ the following keywords were used: *circular economy* AND *model*, *waste* AND *circular economy* OR *waste management*, *Sustainable development goals*, OR *waste production*, OR *waste strategies* OR *environmental management systems*, OR, *KPI indicators* AND *Circular Economy*. All authors participated in the literature review in order to reduce random errors and bias during the research procedure.

The screening of the titles and abstracts for potential inclusion began with consideration of the criteria. Inconsistencies regarding whether a particular study/report/manuscript should be included or excluded were resolved through extensive discussion among the authors. The 2100 Scopus references were cross-checked with Mendeley software (Elsevier 2013, London, United Kingdom) to identify any duplicated studies. When the review team was unsure whether a particular paper met or did not meet the inclusion criteria, full papers were downloaded for further evaluation. The authors collected and analysed data from 101 records, 70 articles and 31 reports related to the topic under consideration. The final records used in the manuscript were agreed upon by all of the authors.

## Results and discussion

A circular economy model is critical for achieving waste management goals and targets through new and innovative business strategies (Ko et al., 2020). Circular economy strategy is the main driving force of a new period of improved waste management and prevention efficiency across the different sectors (Moktadir et al., 2020; Zoboli et al., 2020). Defining a baseline through assessment is crucial for determining what needs to change in order to achieve circular economy targets and objectives. Measuring progress against a baseline over time could assist in achieving widespread and system-wide change. The lack of a systematic structure and methodologies for assessing the circular economy tends to leave uncertainty concerning the efforts and unaware of potential areas for utilization. These constraints also inhibit progress against greater data collection, evaluation and interaction. Several researchers highlight that the existing lack of visibility of accurate data and measurement in circular processes related with waste generation and management cause a gap in knowledge concerning how the circular economy is progressing (Oluleye et al., 2023, 2022; Rincón-Moreno et al., 2021; Spišáková et al., 2022).

The role of KPIs in evaluating circularity performance within waste management could provide valuable knowledge for decision-making methodologies (Colasante et al., 2022; Pires and Martinho, 2019). KPIs are computational sets which try to measure, simplify and communicate data that is difficult to observe unequivocally. They can assess and optimize an establishment’s progress towards a specific goal (Papamichael et al., 2023).

According to Loizia et al. (2019) among the most applicable and suitable, well-known KPIs to assess the level of circularity in waste management are Municipal Solid Waste Production, Waste Composition, Municipal Solid Waste Recycling, Waste Recovery Index, Recycle Bins per Population, Clean Index, Accumulation Rate, Waste Accumulation Index and Waste Generation Rate (Table 1).

**Table 1.** KPIs assessing waste in the context of circularity.

Indicator	Description	Equation
MSW-C	Gives information about the waste streams created during a certain time period	$C-\mathrm{MSW}=\frac{Q_{\mathrm{Known}\;\mathrm{MSW}}t}{Q_{\mathrm{Total}\;\mathrm{MSW}}t}$
MSW-P	Represent the ratio of the amount of MSW produced to the population at a given time	$\mathrm{MSW}-P=\;\frac{Q_{\mathrm{Total}\;\mathrm{MSW}}t}{Q_{\mathrm{POP}}t}$
MSW-R	Compares the amount of recycled MSW to the total amount of MSW generated at a given time	$\mathrm{MSW}-R=\;\frac{Q_{\mathrm{Recycled}\;\mathrm{MSW}}t}{Q_{\mathrm{Total}\;\mathrm{MSW}}t}$
AR	Used to calculate the amount of waste litter accumulated by an item per unit of surface and per unit of time	AR=NIcol/S/T
AI	Takes into account the accumulation rates of the waste litter	AI=log10(AR×1,000,000)
WGR	Estimates the generation of waste in the selected area in unit of time	$\begin{array}{l} \mathrm{WGR}=\mathrm{Waste}\;\mathrm{production}\;\mathrm{in}\;\mathrm{one}\;\mathrm{area}\;\mathrm{in}\;\mathrm{kg}\times \\ \mathrm{Citizens}\;\mathrm{in}\;\mathrm{the}\;\mathrm{same}\;\mathrm{area}\;\mathrm{in}\;1\;\mathrm{day} \end{array}$
WRI	Indicates the recovery of generated waste in a specific period	$\mathrm{WRI}=\frac{\mathrm{Recoverd}\;\mathrm{waste}}{\mathrm{MSW}-P}$
RBP	Estimates the number of recycle bins per population density	$\mathrm{RBP}=\frac{\mathrm{Number}\;\mathrm{of}\;\mathrm{recycle}\;\mathrm{bins}}{\mathrm{Population}\;\mathrm{density}}$
CI	Rates how clean the selected area	$\mathrm{CCI}=\frac{\mathrm{NIcol}}{\mathrm{Sur}}K$

KPI: key performance indicator; MSW-P: Municipal Solid Waste Production; MSW-C: Municipal Solid Waste Composition; MSW-R: Municipal Solid Waste Recycling; WRI: Waste Recovery Index; RBP: Recycle Bins per Population; CI: Clean Index; AR: Accumulation Rate; AI: Waste Accumulation Index; WGR: Waste Generation Rate.

Developing circular economy indicator metrics does not come without obstacles. One of the main concerns seems to be that, while the transition to a circular economy is strongly related to Europe’s climate agenda and SGDs, a focus on materials would not imitate all environmental issues, rendering to the 2030 Agenda for Sustainable Development. Even though underappreciated, the use of KPIs and targets will be vital. Admittedly, ‘what you can’t measure, you can’t manage’. Until 2019, there have been numerous attempts to create waste metrics in the framework of circular economy to be used in the private or public sectors. Despite these remarkable initiatives, there has yet to be an aligned metrics research area (D’Adamo et al., 2022). Whenever it comes to indicator development, interested parties are still speaking ‘many languages’. In order to provide an effective support for the transition to a circular economy, indicators must be coherent, relevant, widely recognized and simple to use. With a number of issues ranging from inherent restrictions in indicator coverage to data accessibility and selection, the circular economy indicator field is still in its early stages and is not yet ready to completely the ability to speed up circular economy adoption (Corona et al., 2019; European Commission, 2018).

Taking into consideration the above obstacles, the stakeholders and policymakers, both at Global and European level, proceeded to develop specific frameworks with targeted indicators to measure the circular economy, including specific indicators for waste production management. Except those efforts, additionally initiatives from national and private sector have been implemented in an attempt to capture circular economy progress (Table 2).

**Table 2.** Existing initiatives measuring circular economy (European Commission, 2018).

Level	Title	Description
World /EU level	EU Monitoring Framework for the Circular Economy (2018)	10 indicators related with production, consumption, waste management, raw materials, etc.
	EU Resource Efficiency Scoreboard (2013)	A set of resource efficiency indicators that includes a lead indicator for resources, dashboard indicators for materials, water carbon, as well as theme-specific indicators
	Raw Materials Scoreboard (2016)	Indicators related to the EIP on raw materials, providing quantitative data to the EU on the EIP’s objectives and natural resources in the context of EU policy
	OECD (2021)	The OECD inventory of circular economy with 474 indicators
	UNEP (2013)	10 KPIs
	GRI (2016)	>100 indicators
	World Bank	50 indicators related with environment and sustainability
	The Global Platform for Accelerating Circular Economy (Sitra, 2018)	Presents their recommendations for improving circular indicators and measurement
	Bellagio Declaration (2021)	Includes a set of seven principles that describe the essential components of a circular economy monitoring framework
	Australia (2022)	3 Indicators groups for circular economy measurement
National	China (2017)	The national indicator system in China is focused on material flow accounting involves 17 indicators
	France (2017)	10 indicators, monitor the circularity of the French economy. It comprises a comparison to the EU
	Netherlands (2018) (Potting et al., 2018)	21 indicators to measure circularity in the Netherlands
	Japan (2013)	Indicators based on economic material flow dimensions (input, circulation and output)
	Germany (2016)	Indicators to measure the sustainable use and conservation of raw material
	Spain (2017)	20 indicators assessing circularity
	Belgium (2015)	21 measures to be followed
Private	Circularity Gap Report (2018)	As a single metric of circular economy, the Global Circularity Metric based on the percentage of cyclical use of natural resources is recommended
	Ellen MacArthur Foundation Circularity Indicators (2015)	Economic activities and merchandise tools for measuring circularity. The main indicator is related with resource circularity.
	University of Cambridge Circular Economy Toolkit	Based on quantitative survey data, an online self-assessment tool for companies serves as a guide
	Circle Economy Circle Assessment (2017)	Online business tool that focuses on seven factors to enhance organizational activities, aid in the implementation of circular economy strategies at a business level

KPI: key performance indicator; EU: European Union; GRI: global reporting initiative; EIP: European Innovation Partnership; OECD: Organisation for Economic Co-operation and Development; UNEP: United Nations Environment Programme.

Waste metrics in the framework of circular economy are commonly consolidated at EU and national levels by statistical offices, who use relevant information from economy-wide material flow accounts, economic and financial data and waste statistics (such as waste production, recycling rates, etc.). At the same time, when integrated into circular economy, such metrics can aid with the core principles of circularity such as resource monitoring and efficiency (extension of products lifespan), the reduction of environmental impacts, economic benefits and new economic opportunities (based on data and not assumptions), as well as innovation potential (Ellen MacArthur Foundation, 2021). These methods aid in giving an overall picture of the material intensity of a specific economy or sector, considering both residential consumption of raw materials and products export and import. However, MFA is unable to provide a sufficient amount of information for entrepreneurs and their products at the national and sectoral analysis levels due to the massive volume of data that must be applied, as well as the need to cope with uncertainties (Millette et al., 2019). Organization or product-specific metrics were also established in order to track material flows across supply chain analysis, leveraging tools such as LCA. Such business metrics tend to regard key aspects underlying the circular economy, such as designing products, partnership models and reverse logistics, which can be measured quantitatively or qualitatively. The Ellen MacArthur Foundation (EMF) and Granta Design, for example, use the concept of ‘Material Circularity Indicator’ to focus on MFA in a business or product (European Commission, 2018).

One of the most concerted efforts to develop a widely accepted framework for measuring circular economy was the Platform for Accelerating the Circular Economy (PACE) published in 2018 (IISD, 2018; Sitra, 2018). PACE was a joint effort from the World Economic Forum, UNEP, World Resources Institute, Philips, EMF and 40 other partners. The initial aim of PACE was threefold: (i) developing society and economic models for circular economy initiatives, particularly in developing countries, (ii) developing strategy frameworks that address identified barriers to expanding the circular economy and (iii) enhance public collaborations for all these purposes.

Over the last couple of years, China has prioritized the concept of the circular economy as a national regulatory policy priority. This enhanced the circular economy principle at three different levels: the independent firm, the eco-industrial park and the eco-province level. Circular economy mainly includes eco-design and greener production strategies at the firm level (Papamichael et al., 2022a). Eco-industrial parks and networks with beneficial effects both on the local economy and on the environment are endorsed at the meso-level, whereas at the macro/national level, both sustainable production and consumption practices are encouraged with the objective of developing a recycling-oriented civilization (EASAC, 2015, 2016). In 2021, the National Development and Reform Commission launched the Development Plan for the Circular Economy in the 14th Five Year Plan Period. The Plan intends to promote the circular economy through a number of measures including recycling, remanufacturing, green product design and renewable resources. The Plan establishes a set of specific numerical goals for the government to meet by 2025. These include the following: (i) increasing resource productivity by 20% over 2020 levels; (ii) decreasing energy and water consumption per unit of GDP by 13.5% and 16%, respectively, over 2020 levels; (iii) achieving a utilization rate of 86% for crop stalks, 60% for bulk solid waste and 60% for construction waste; (iv) utilizing 60 million tonnes of paper waste and 320 million tonnes of scrap steel (v) generating 20 million tonnes of recycled nonferrous metals and (vi) raising the overall value of the resource recycling industry to RMB 5 trillion (US$773 billion) (Bleischwitz et al., 2022).

With the Law for Promotion of Effective Utilization of Resources adopted by Japanese Government, Japan established a basic legislative framework for empowering circular economy 25 years ago (1991). A Law for Establishing a Material-cycling Society (2000) applied to recycling of automobiles, packaging waste, domestic appliances, demolition waste and food wastes. Extensive and consumer-friendly refund systems, including prepayment for return and recycling at the point of sale as well as cooperation among producers in recycling (United Nations Environment Programme, 2017).

An Australian circular economy measurement framework included headline, impact, and transition indicators that record different aspects of the circular economy. The following measurement framework targets also defined communication, priority setting, tracking and correlative performance metrics throughout cities and counties, as well as an acceptable indicator hierarchy (IISD, 2018).

The headline indicators provide a high-level index about how circular economy represented by percentage or resources consumed per unit, the impact indicators examine the effect of consumption of resources, including the economy, environment and society, and the transition indicators examine how the circular economy is tracking against makers of progress towards the future state (IISD, 2018).

Another important step for circular economy measurement and evaluation were the models that used a set of steps or levels or strategies of circular economy, commonly beginning with the letter ‘R’ in English. The first one, widely recognized as the ‘Three R principle’, was ‘Reduce, Reuse, Recycle’, which dates back to the 1970s. Similarly, Ellen MacArthur Foundation (2015) and Lansik in 2015 proposed their R strategies. However, according to Breteler (2022), the ‘10R principle’, established by Professor Cramer in 2017, is the ‘greatest extensive’ of the four compared models presented in the Table 3 (Cramer, 2022).

**Table 3.** Levels of circularity (Breteler, 2022).

10R principle (Cramer. 2017)	Ellen MacArthur Foundation (2015)	Ladder of Lansink (Lansink, 2015)	Three R principle (1970s)	Explanation (Cramer, 2017)
Refuse	Maintain/prolong	Prevention	Reduce	‘Prevent raw materials use’
Reduce				‘Decrease raw materials use’
Renew/Redesign				‘Redesign product in view of circularity’
Reuse	Reuse/redistribute	Reuse	Reuse	‘Use product again (second hand)’
Repair				‘Maintain and repair product’
Refurbish	Refurbish/remanufacture			‘Revive product’
Remanufacture				‘Make new product from second hand’
Repurpose				‘Re-use product but with other function’
Recycle	Recycle	Recycling	Recycle	‘Salvage material streams with highest possible value’
Recover	Energy recovery	Energy recovery		‘Incinerate waste with energy recovery’
		Incineration		
	Landfill	Landfill		

Even though, the initial emphasis of research community, business and governance was mainly on the creation of re-X strategies (recycling, remanufacturing, reuse, etc.) it soon became apparent that advanced technologies progressively exceeded their implementation. Multiple stakeholders work collaboratively to utilize this technology for the transition to a circular economy. This turned its attention to business-model innovation as a crucial lever for circular technology adoption (Papamichael et al., 2022a). Rheaply is an example of a new tech that puts an emphasis on investment management and disposition that enables organizations transitioning to circular economy business model (Solve, n.d.). Circular economy business models, close, narrow, slow, intensify and dematerialize loops in order to reduce materials inputs into and waste leakage from the organizational system. This includes recycling metrics, productivity improvements, life cycle extensions and product substitution with software and service alternatives (Papamichael et al., 2023a). The above strategies could be noticed through the creative design of resources recovery methods and associated circular supply chains (Batista et al., 2019). Circular business models could have distinct differences and targets, such as extending the life cycle of products over numerous ‘use cycles’; use a ‘waste = food’ approach in order to assist products recovery ensuring that biological materials brought back to the earth are not harmful; keep embedded inputs such as energy and water in the product as longer as possible; design solutions using systems-thinking initiatives; promote product stewardship through policy initiatives, taxation and market mechanisms such as ‘polluter pays’ regulations (Weetman, 2016).

To strengthen the circular model, the scientific and research community developed specific standards with specific requirements. In 2017, the British Standards Institution developed and published the first circular economy standard, ‘BS 8001:2017 Framework for implementing the principles of the circular economy in organizations’, to provide credible guidance to organizations implementing circular economy strategies (BSI, 2017). The standard BS 8001:2017 attempts to harmonize the circular economy’s ambitious goals with existing practices at the organization level. It includes an extensive reference guide of circular economy terms and definitions, a characterization of the fundamental circular economy principles and an adjustable management plan for implementing circular economy approaches in organizations. However, there is limited concrete guidance on circular economy evaluation and monitoring due to the lack of agreement on a set of core circular economy KPIs applicable to both organizations and individual products (Pauliuk, 2018).

In 2018, the International Organization for Standardization (ISO) developed a technical committee (TC 323) in the sector of circular economy to generate frameworks, guidelines and specifications for the execution of initiatives by all relevant stakeholders in order to increase their contribution to SDGs. Six new ISO standards are currently being developed under the direct supervision of this technical committee: (i) ISO/CD 59004 Circular Economy – Terminology, Principles and Guidance for Implementation; (ii) ISO/CD 59010 Circular Economy – Guidance on the transition of business models and value networks; (iii) ISO/CD 59020 Circular Economy – measuring and assessing circularity; (iv) ISO/CD TR 59031 Circular economy – Performance-based approach – Analysis of cases studies; (v) ISO/CD TR 59032.2 Circular economy – Review of business model implementation and (vi) ISO/WD 59040.2 Circular Economy – Product Circularity Data Sheet (ISO, 2018).

In addition to the above initiative, digitalization and digital technologies (e.g. blockchain, big data, IoTs, artificial intelligence, etc.) are considered to be core elements for scaling up the circular economy. The key role of digital technologies in speeding up the circular economy transition, also known as the data economy, is underlined with the EGD (Çetin et al., 2022). The smart circular economy approach embodies this by connecting digital technologies to sustainable management of resources. This enables for the evaluation of various digital circular economy strategies and guidance on how to use data and analytics to boost circularity (Kristoffersen et al., 2020; Sadeghi et al., 2022).

As automation and real-time monitoring help to integrate circular economy into daily operations, the adoption of digitalization has the potential to minimize resource usage. The concepts of digitization, circular economy and sustainable development may be used as principles for managing uncertainty in product development processes. In order to facilitate informed decision-making, digitization is being utilized in many businesses and industries for accounting administration (Varaniūtė et al., 2022).

Alonso et al. (2021), highlighted how automating recycling processes through the use of convolutional neural networks and deep learning techniques may help in detecting and categorizing recyclables through photographs. These networks used images as input and used feature detection to discriminate between various materials. In addition, current techniques like TRashNet or MobileNet V2 can categorize materials using datasets and have a classification accuracy of 97% (Melinte et al., 2020). Similar results are obtained by VGG16, GoogleNet and other networks in the categorization of specific materials (such as paper, plastic, glass, metal, etc.) (Alonso et al., 2021).

In order though for circular economy models to be implemented and quantified, several incentives need to be in place for all major players, including but not limited to policymakers, producers, urban planners and the public. Offering financial incentives, such as tax breaks, subsidies or grants, and other regulatory relief measures, can encourage businesses and individuals to adopt circular practices. These incentives can be targeted towards activities such as resource recycling, product repair or the development of innovative circular business models (Papamichael et al., 2022a). At the same time, the extended producer responsibility (EPR) policies shift the responsibility for managing the end-of-life of products onto the producers. By making manufacturers accountable for the entire lifecycle of their products, EPR incentivizes them to design products for durability, recyclability and ease of repair. EPR programs can include regulations, financial incentives and recycling targets (Loizia et al., 2021). Furthermore, key aspect of motivation towards adequate circular economy models is public procurement, where governments can use their purchasing power to drive demand for circular products and services. By incorporating circularity criteria into public procurement processes, alongside with monitoring tools, governments can create a market for circular solutions and encourage businesses to develop innovative circular products and services (Pappas et al. 2022). Key element of such shift is the targeted and substantial education and transfer of knowledge to the public. Raising awareness about the benefits of the circular economy and educating individuals and businesses about circular practices can be powerful motivators. Campaigns can highlight the environmental, economic and social advantages of circularity, inspiring behaviour change and encouraging adoption of circular practices (Eliades et al., 2022).

In addition to encouraging circular economy initiatives in the commercial and industrial sectors, policymakers may also embrace digital transformation to boost sustainability and the economy (Barteková and Börkey, 2022). According to the European Environment Agency (2021), ‘MarineLItterWatch’ mobile application combines online platforms and databases to connect marine litter on beaches reported by citizens. In the subject matter of circular economy, ‘Circulytics’ tool developed by the Ellen MacArthur Foundation helps businesses make a shift towards circular economy by measuring the circularity of an organization, supporting decision-making and the creation of strategic planning. Also this tool is used for identifying blind spots and strengths and providing clarity for the adoption of circular economy from the investors and customers (Ellen MacArthur Foundation, n.d.). The interconnection of digitalization with existing monitoring tools (i.e. KPIs) to measure circularity in different settings (i.e. business, city, nation, etc.) can provide a more efficient route towards circularity.

## Conclusion

The circular economy does not seek to disrupt profit maximization paradigms in enterprises. Instead, it offers an alternative approach to maintaining a competitive advantage while addressing the environmental and socioeconomic challenges of the 21st century. Shifting away from linear production models often leads to the development of new internal capabilities across the value chain, resulting in increased efficiency, cost reduction, enhanced productivity, brand enhancement, risk mitigation, product innovation and compliance with regulations and green consumer expectations. The integration of digital technologies, such as artificial intelligence, blockchain and the IoT, can help overcome barriers and facilitate the transition to a circular economy, while also significantly influencing consumption and production patterns in today’s society. Harnessing the benefits of Industry 4.0 can have substantial positive effects on the pillars of sustainability (environment, society, economy). For the successful implementation and quantification of circular economy models, it is necessary to establish various incentives for all key stakeholders, including policymakers, producers, urban planners and the public. These incentives should encompass a range of measures, including but not limited to financial incentives such as tax breaks, subsidies and grants, as well as regulatory relief. By providing such incentives, businesses and individuals can be motivated to embrace circular practices. However, monitoring alone will not suffice for a seamless transition; it is crucial to involve all relevant stakeholders. To improve quality of life and minimize the human impact on the environment, future efforts must promote a hybrid approach that combines monitoring the circular economy with citizen participation and public awareness.
